# Optimization of external container delivery and pickup scheduling based on appointment mechanism

**DOI:** 10.1371/journal.pone.0318606

**Published:** 2025-02-21

**Authors:** Pengfei Huang, Hao Wang, Fangjiao Tan, Yuyue Jiang, Jinfen Cai

**Affiliations:** Navigation College, Jimei University, Xiamen, Fujian, China; Chouaib Doukkali University: Universite Chouaib Doukkali, MOROCCO

## Abstract

Port transport efficiency has become an urgent issue that needs to be improved, especially the coordination among truck drivers during peak hours. Previous studies mainly focus on one-way container transportation logistics issues, but container movements often occur simultaneously in both directions in practice. Therefore, this study aims to minimize truck companies’ operational costs by establishing an optimization model for external truck scheduling. This model takes soft time windows and an appointment feedback mechanism into consideration. Building upon the traditional Ant Colony Optimization (ACO) algorithm, this paper introduces an adaptive version of the ACO algorithm. The improved Ant Colony Optimization algorithm (IACO) incorporates a time window width impact factor and a time deviation consideration into its state transition rules, enhancing its adaptability. Furthermore, by integrating Particle Swarm Optimization (PSO), the algorithm intelligently tunes the pheromone and heuristic factors of ACO, achieving automatic parameter optimization. Through case studies, we have demonstrated the superior performance of this algorithm in addressing relevant problems. The results show that, in terms of truck operational costs, the improved algorithm reduces costs by 10.96% and 3.02% compared to traditional Ant Colony Optimization and Variable Neighborhood Search algorithms, respectively, and by 4.89% compared to manual scheduling. These results demonstrate that the adaptive Ant Colony Optimization algorithm exhibits clear advantages in optimization capability and stability. The algorithm effectively allocates truck tasks within each time window, thereby reducing fleet costs, improving truck turnover efficiency, mitigating port congestion, and ultimately enhancing container logistics efficiency, achieving the goals of peak shaving and valley filling.

## Introduction

Container transportation, as a primary mode of maritime transport, has experienced significant growth due to its standardized, high-density, and efficient characteristics. Nowadays, a large terminal handles millions of containers annually [1]. The increase in container throughput means that the port needs to handle more cargo within the same time frame, which places higher demands on the port’s container transportation efficiency. Container trucks, as the main transportation mode between the port and external yards, can be divided into internal and external trucks based on their operational range. Internal trucks primarily handle container transportation within the port, while external trucks are responsible for container transportation between the port and inland areas. Compared to internal trucks, external trucks operate over a broader range, and their scheduling efficiency directly impacts the port’s overall operational costs, container transfer efficiency, and the functioning of the entire logistics system.

Due to factors such as the uncertainty of vessel arrival times and fluctuations in road traffic conditions, there is often a situation where a large number of trucks arrive at the port simultaneously during peak hours [2]. Due to the limited port capacity, it is difficult to efficiently process all arriving trucks within a short time. Trucks are forced to wait in long queues both inside and outside the port, leading to a significant decrease in port operation efficiency. This not only prolongs the time containers spend at the port but also prolongs the high-load operation of the yard cranes [3].In response to this challenge, considering the long construction period and high investment required for port expansion, the Truck Appointment System (TAS) has emerged as an effective solution [4]. The system can avoid a large number of trucks concentrating at the port during peak hours by scheduling and accurately allocating truck arrival times in advance, thus reducing port congestion to some extent and improving container handling efficiency [5]. For instance, the successful implementation of the TAS at the Port of Vancouver has demonstrated its significant effectiveness in optimizing port operations and enhancing transportation efficiency [6].

The TAS System is an integrated management system composed of the external truck scheduling system, internal operations system, comprehensive information management system, and collaborative scheduling system. Among these components, the external truck scheduling system plays a crucial role in optimizing the scheduling order and arrival times of container trucks, thereby enhancing port container handling efficiency and reducing truck fleet operational costs.

Research on external truck scheduling systems at home and abroad can be broadly classified into three categories: (1) TAS system [7–9]; (2) Queuing system at the gate [10,11]; (3) Time-Dependent pricing [12,13].

In terms of TAS, existing research primarily focuses on optimizing the scheduling of off-terminal trucks for different appointment periods. For example, Huynh et al. [14] studied the impact of truck appointment systems on truck turnaround time and crane utilization at container terminals. They proposed a methodological approach through mathematical modeling to assist terminal operators in determining the optimal number of trucks to be accepted within the appointment system. He et al. [15] considered constraints related to truck loading capacity and ARMG mobile operations, maintaining application balance between different periods and stages while optimizing truck allocations for each period. Ramirez et al. [16] considered the impact of the TAS system on terminal yard operations, aiming to reduce container rehandling and truck turnover time, using discrete event system modeling and solving with heuristic algorithms. Ahmed Azab et al. [17] in order to address the issue of trucks spending excessive time waiting at the terminal, combined the actual situation of transportation companies and terminals, and proposed a simulation optimization method aimed at reducing the total turnover time of trucks. Most of these methods focus on optimizing the scheduling of the external trucks delivery process from the perspective of terminal operations, without considering the interests of coordinating truck companies.

Regarding research on gate queue management, some scholars have focused on optimizing the waiting time of external trucks at terminal gates or altering queuing rules to adjust truck arrival shares across different periods, thereby maximizing gate resource utilization. Dekker R et al. [18] proposed establishing a Chassis Exchange Terminal (CET) as an offshore terminal, where CET and the terminal jointly handle chassis exchange during peak periods and perform container transportation during off-peak periods. This approach aims to reduce the burden on terminal gates and improve overall operational efficiency. Zhang et al. [19] developed a BCMP queuing network model to describe the queuing process of trucks at the terminal and designed a method based on Genetic Algorithm (GA) and Point Steady-State Fluid Flow Approximation (PSFFA). Maurício et al. [20] employed data mining techniques, using key performance indicators such as the average and maximum waiting times of trucks at entry gates and the percentage of missed appointments to balance truck arrivals across time windows and reduce waiting times, designing the PFFA algorithm to calculate truck waiting times. Chen et al. [21] considered the impact of arriving vessels on truck fleets. Based on the theory of Variable Delivery Time Windows (VDTWs), they proposed a method of controlling truck arrival times using “vessel-associated time windows.” By integrating queueing theory, they established an optimization model for port arrival time windows and solved it using a hybrid genetic algorithm. However, limited terminal land resources and the potential for terminal yard congestion due to a large influx of trucks passing through the gates in a short period remain challenges.

Some researchers have addressed congestion issues by implementing additional charges during peak periods. For example, Chen et al. [22] proposed congestion relief fees for each period to reduce truck delays and congestion during peak hours. The PierPass program, initially introduced in 2005 by the West Coast Marine Terminal Operators Agreement (WCMTOA), charges a peak period fee for container trucks during weekday afternoons (3 p.m. to 6 p.m.), aiming to shift cargo handling to off-peak periods and thereby distribute traffic more evenly. Although peak period fees have alleviated congestion to some extent, they have also resulted in losses for cargo owners and truck companies. While this paper does not employ such methods, it establishes maximum acceptance capacities and flexible time windows for terminal gates, encouraging trucks to avoid peak times and arrive punctually.

Currently, most research on the scheduling between external yards and terminals has focused on one-way transportation, often emphasizing the interests of terminal operators. The main objectives of such studies have been to minimize truck turnaround time or reduce terminal operating costs. Additionally, traditional methods typically treat time windows as rigid constraints, making them difficult to adapt to the complexities of real-world scenarios, which results in lower flexibility in scheduling solutions. Furthermore, traditional approaches such as Ant Colony Optimization (ACO) often rely on empirically set parameters, which cannot dynamically adjust to meet the varying needs of different problems, thus limiting the algorithm’s solving efficiency and the quality of the solution.

To address these issues, this study proposes a truck scheduling model for external yards based on an appointment system, with the objective of minimizing fleet operational costs. An improved Ant Colony Optimization (IACO) algorithm is designed, which optimizes the pheromone matrix and state transition formulas. The model introduces time deviations and time window widths, enhancing the adaptability and optimization capability of the scheduling. Moreover, by integrating Particle Swarm Optimization (PSO) to dynamically search for the key parameters of the Ant Colony Algorithm, the model achieves adaptive parameter adjustment, significantly improving the algorithm’s convergence efficiency and solution quality.

## Problem description and formulation

### Problem description

This study investigates the bidirectional scheduling and transportation between multiple external yards and a single terminal, incorporating soft time windows and penalty mechanisms to flexibly adjust the internal operation sequences of container trucks, thereby reducing operational costs for trucking companies.

**Fig 1:**
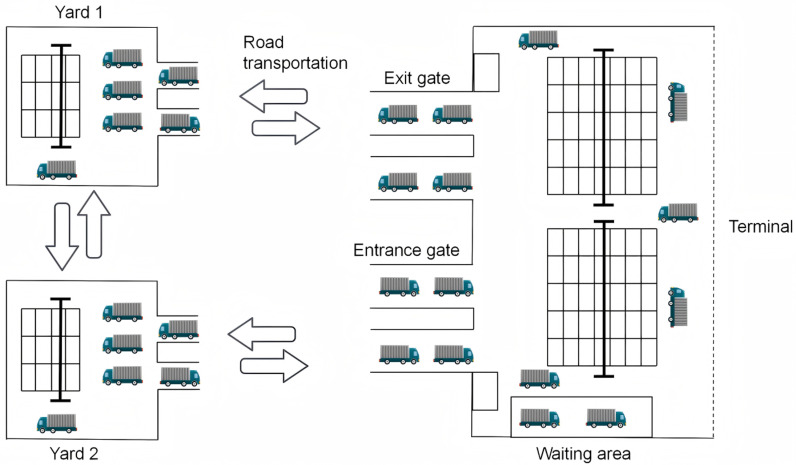
The layout of the external truck collection and distribution operation.

External truck scheduling is a key link in port logistics, influenced by various complex factors. The primary factors include gate throughput efficiency, time window duration, traffic conditions, and yard loading and unloading efficiency. As a result, the round-trip time of external truck transportation operations is uncertain, making it difficult to predict the arrival time and volume of trucks at the port, which further complicates the scheduling process. Fig 1 illustrates the layout of the external truck collection and distribution operation system at the port.

Due to the uncertainty in container truck arrival times, during peak hours, the concentration of a large number of trucks arriving at the port can cause traffic congestion in the port area, overload yards, operating equipment, and reduce overall operational efficiency. In contrast, during off-peak hours, port resources may remain underutilized, leading to low operational efficiency and increased idle costs. By introducing the TAS, the 24-hour day is divided into different time slots. The port can set the time window width and the maximum number of containers to be handled in each time slot based on factors such as the operational capacity of each period, yard space, and the number of import and export containers. This ensures balanced utilization of port resources and improves overall operational efficiency.

**Fig 2:**
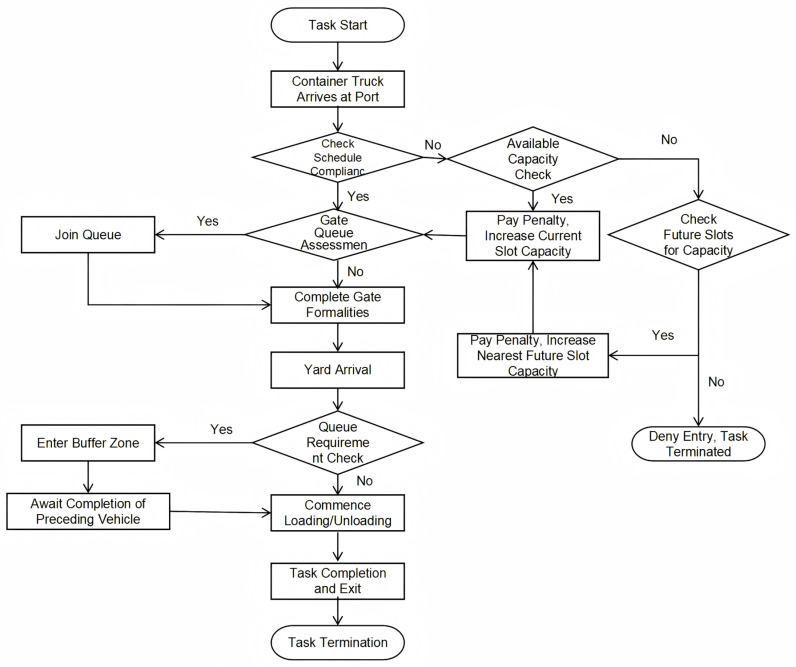
Schematic diagram of the process for booking container trucks to enter the port.

Trucking companies optimize the internal operation sequences of container trucks according to the scheduling plan prepared by the terminal. The trucks complete the loading and unloading operations within the designated time frame, thereby reducing operational costs. The process of truck arrival at the port based on the TAS system is shown in Fig 2:

The TAS system continuously provides feedback and makes adjustments, balancing the number of trucks arriving at the port during each time period through the use of soft time windows, optimizing the container transport operation process, and reducing truck waiting times at the port, which in turn alleviates yard congestion. This system’s coordination and optimization not only improve the overall operational efficiency of the port but also help trucking companies reduce empty load rates and operational costs.

Building upon this, this paper takes the scenario of a single terminal and multiple external yards for container pickup and delivery as a background. By integrating soft time windows and penalty mechanisms, it develops an external truck scheduling optimization model based on a reservation feedback mechanism. The main goal of this model is to minimize the overall cost of the fleet while completing the transport tasks, from the perspective of optimizing the transportation quota for each time period. To achieve this, an IACO algorithm is designed to solve the model, aiming to enhance the optimization results and solving efficiency.

### Parameters and variables

For simple tracking, all notations used in the paper are summarized in Table 1.

**Table 1:** Notation list.

Variable	Interpretation	Unit
*J*	Terminal set, *J* = {*j | j* = 1}.	—
*I*	Depots set, *I* = {*i* | *i* = 2, 3, 4, …, I}.	—
*P*	The set of terminal and depots, *P* = {*p* | *p* = 1, 2, 3, …, *P*}.	—
*K*	Set of truck IDs, *K* = {*k* | *k* = 1, 2, 3, …, *K*}.	—
*T*	Set of appointment time periods, *T* = {*t* | *t* = 1, 2, 3, …, *T*}.	—
*N*	The delivery count for the same vehicle within a single time period.	—
*X*_ijntk_	Decision variable *X*_*ijntk*_ is defined as follows: *X*_*ijntk*_ = 1 if truck *k* participates in the *n*-th delivery from depot *i* to terminal *j* during time period *t*; otherwise, *X*_*ijntk*_ = 0.	—
*R*_t_	The maximum number of trucks that the terminal can accept during the time period *t*.	—
*Y*	Decision variable *Y* is defined as follows: *Y* = 1 when there is overload transportation, and *Y* = 0 otherwise.	—
*K*_ip_ *, K*_pi_	Represent the number of trucks departing from and returning to the depots respectively.	—
*D*_ij_	Distance from depot i to terminal *j*	km
*t*_1_	Average handling time at the depots.	min
*t*_2_	Average waiting time at the terminal gate.	min
*t*_3_	Terminal operation time.	min
*C*_k_	The sum of daily labor costs and truck wear and tear costs.	RMB
*C*_l_	Fuel cost at full load.	RMB/km
*C*_e_	Fuel cost at empty load.	RMB/km
*C*_i_	Idle fuel consumption cost.	RMB/h
*C*_p_	Penalty fee for trucks passing through the gate.	RMB
*T*_a_	Arrival time of container delivery trucks at the terminal.	min
*t* ^r^_ij_ *, t* ^d^_ij_	The scheduled start and end times for container delivery from depot *i* to terminal *j*.	min
*t*_*e*_ *, t*_*l*_	Earliest and latest acceptable times relative to the appointment, representing the front and back ends of the flexible time window.	min
*p*_1_ *, p*_2_	Penalty factors for arriving before and after the scheduled appointment time.	—
*Q*_ij_	The total quantity of container deliveries from depot *i* to terminal *j*.	—
*Q*_ji_	The total quantity of container pickup from terminal *j* to depot *i*.	—
*T*_jintk_	The completion time of truck *k* delivering the *n*-th container from terminal *j* to depot *i* during time period *t*.	min
*T*_ijntk_	The completion time of truck *k* delivering the *n*-th container from depot *i* to terminal *j* during time period *t*.	min
*T*_ipn’t’k_	Next container delivery start time.	min
*t*_v_	Travel time from the depot to the terminal.	min
*t*_w_	Depot waiting time.	min

### Assumptions

Several model settings and assumptions are outlined as follows to better clarify the problem:

(1) Each reservation period is set to two hours, with the total container volume as well as the reservation quotas for each period being predetermined.
(2) The terminal only accepts container collection tasks that are scheduled within the specified time frame; tasks without a reservation or outside the designated period are not accepted. Container distribution tasks are not constrained by reservation periods but must be completed within the stipulated timeframe.
(3) The distances between the terminal and each depot are fixed and unaffected by traffic conditions, assuming that all drayages arrive on time.
(4) Drayage transport time includes both empty and loaded transport times, determined by the ratio of the distance between locations and the respective speeds.
(5) Yard operation time includes waiting and loading/unloading times.
(6) Terminal operation time includes gate waiting times, waiting area waiting times, loading/unloading times, and internal driving times within the terminal; the average terminal operation time is used to represent waiting area waiting times, loading/unloading times, and internal driving times, which vary across terminals.
(7) Each container drayage truck can only carry one standard container of the same specification, without considering other container types. Besides, all performance indicators for container drayage trucks are assumed to be identical.

### Model construction

This paper constructs a model for the simultaneous pickup and delivery operations of external container trucks under the mechanism of appointment, with the condition of soft time windows. The objective is to minimize the fleet operation cost, where the delivery objective function is shown in Eq (1).


$$Z_{\min}=Z_{1}+Z_{2}+Z_{3}+Z_{4}$$ (1)

$${\text{Z}}_{\text{1}}\text{=}\sum_{\text{i=2}}^{\text{I}}\sum_{\text{j=1}}^{\text{J}}\sum_{\text{n=1}}^{\text{N}}\sum_{\text{t=1}}^{\text{T}}\sum_{\text{k=1}}^{\text{K}}\left\{{\text{X}}_{\text{ijntk}}\left[\frac{\left({\text{t}}_{\text{1}}{\text{+t}}_{\text{3}}\right){\text{C}}_{\text{i}}{\text{+2t}}_{\text{2}}}{\text{120}}{\text{+YD}}_{\text{ij}}{\text{C}}_{\text{1}}{\text{+(1-Y)D}}_{\text{ij}}{\text{C}}_{\text{e}}\right]{\text{+X}}_{\text{jintk}}\left[\frac{\left({\text{t}}_{\text{1}}{\text{+t}}_{\text{3}}\right){\text{C}}_{\text{i}}}{\text{120}}{\text{+YD}}_{\text{ji}}{\text{C}}_{\text{1}}{\text{+(1-Y)D}}_{\text{ji}}{\text{C}}_{\text{e}}\right]\right\}$$ (2)

$$Z_{2}=\sum_{i=2}^{I}\sum_{i=2}^{I}\sum_{t=1}^{T}\sum_{k=1}^{K}X_{iint\text{k}}\left(\frac{t_{1}C_{i}}{60}+\frac{D_{ii}C_{e}}{V_{e}}\right)$$ (3)

$$Z_{3}=\sum_{k=1}^{K}C_{k}$$ (4)

$$Z_{4}=\sum_{i=2}^{I}\sum_{j=1}^{J}\sum_{n=1}^{N}\sum_{t=1}^{T}\sum_{k=1}^{K}X_{ijntk}C_{p}$$ (5)

$$C_{P}=\{\begin{array}{c} {\text{p}}_{\text{1}}\left({t^{r}}_{ij}-t_{a}\right),{\text{t}}_{\text{a}}\in \left({t^{r}}_{ij}-t_{e},{t^{r}}_{ij}\right)\\ 0,{\text{t}}_{\text{a}}\in \left({t^{r}}_{ij}\right),\left({t^{d}}_{ij}\right)\\ p_{2}\left(t_{a}-{t^{d}}_{ij}\right),{\text{t}}_{\text{a}}\in \left({t^{d}}_{ij},{t^{d}}_{ij}+t_{1}\right) \end{array}$$ (6)

Eq (1) is the objective function, which aims to minimize the total cost composed of transportation costs, transfer costs, fixed costs, and penalty costs. Eqs (2–5) respectively illustrate the specific calculation methods for the aforementioned costs. Specifically, Eq (2) is the transportation costs; Eq (3) represents the cost associated with reallocating idle trucks from one depot to another that requires additional trucks; Eq (4) is the fixed expenses of trucks, including driver costs and wear-and-tear costs of those trucks; Eq (5) is the penalty fees incurred by container delivery trucks passing through the gate, whose details are presented in Eq (6).


$$K_{ip}=K_{pi}$$ (7)

$$\sum_{i=2}^{I}Q_{ij}=\sum_{i=2}^{I}\sum_{j=1}^{J}\sum_{n=1}^{N}\sum_{t=1}^{T}\sum_{k=1}^{K}X_{ijntk}$$ (8)

$$\sum_{i=2}^{I}Q_{\text{ji}}=\sum_{i=2}^{I}\sum_{j=1}^{J}\sum_{n=1}^{N}\sum_{t=1}^{T}\sum_{k=1}^{K}X_{jintk}$$ (9)

$$\sum_{i=2}^{I}\sum_{j=1}^{J}\sum_{n=1}^{N}\sum_{k=1}^{K}X_{ijntk}\leq R_{t}$$ (10)

$$X_{ijntk}\in \{0,1\},X_{jintk}\in \{0,1\}$$ (11)

$$T_{jintk}=T_{ijntk}+\frac{\text{1}}{2}{\text{t}}_{\text{1}}+{\text{t}}_{\text{v}}{\text{+t}}_{\text{2}}\text{+}\frac{\text{1}}{2}{\text{t}}_{\text{3}}$$ (12)

$$T_{ijn't'k}=T_{jintk}+\frac{\text{1}}{2}{\text{t}}_{\text{3}}+{\text{t}}_{\text{v}}{\text{+t}}_{\text{w}}{\text{+t}}_{1}$$ (13)

$$0\leq t_{ir}-t_{e}\leq T_{ijntk}\leq t_{id}+t_{l}\leq 1440$$ (14)

Eqs (7–11) are constraint conditions. Specifically, Eq (7) ensures that all trucks departing from a container depot will ultimately return to the same depot. Eq (8) represents the constraint on the total number of containers delivered from the depot to the terminal, while Eq (9) represents the constraint on the total number of containers picked up at the terminal. Eq (10) constrains the number of trucks arriving at the terminal gate to not exceed its maximum capacity. Eq (11) represents the constraints for 0-1 variables. Eqs (12 and 13) are time constraints, ensuring that the delivery and pickup tasks form a closed loop. Eq (14) ensures that the truck arrival times fall within the terminal’s appointment time window and the flexible time range.

### IACO algorithm design

#### Algorithm flow.

The traditional ACO algorithm simulates the collective intelligence process of ants searching for food through pheromone transmission. The core idea is that multiple ants randomly traverse the solution space, selecting their movement direction based on the pheromone concentration on the path and heuristic information, continuously updating the pheromone concentration to increase the probability of selecting optimal paths. Although traditional ACO has achieved good results in many optimization problems, it is prone to getting stuck in local optima during the early stages, leading to low search efficiency. Additionally, due to the pheromone update mechanism, the algorithm converges slowly and often suffers from premature convergence. Therefore, traditional ACO has certain limitations when solving complex problems.

To overcome these issues, this paper proposes an IACO algorithm to solve the model. The improved algorithm introduces an adaptive factor in the pheromone update process, which strengthens the early-stage search and prevents premature convergence. Additionally, the algorithm incorporates an elitist ant system to ensure that the pheromone on the optimal path is reinforced in a timely manner. PSO is also used to automatically tune the algorithm’s parameters, further reducing the likelihood of local optima.

**Fig 3:**
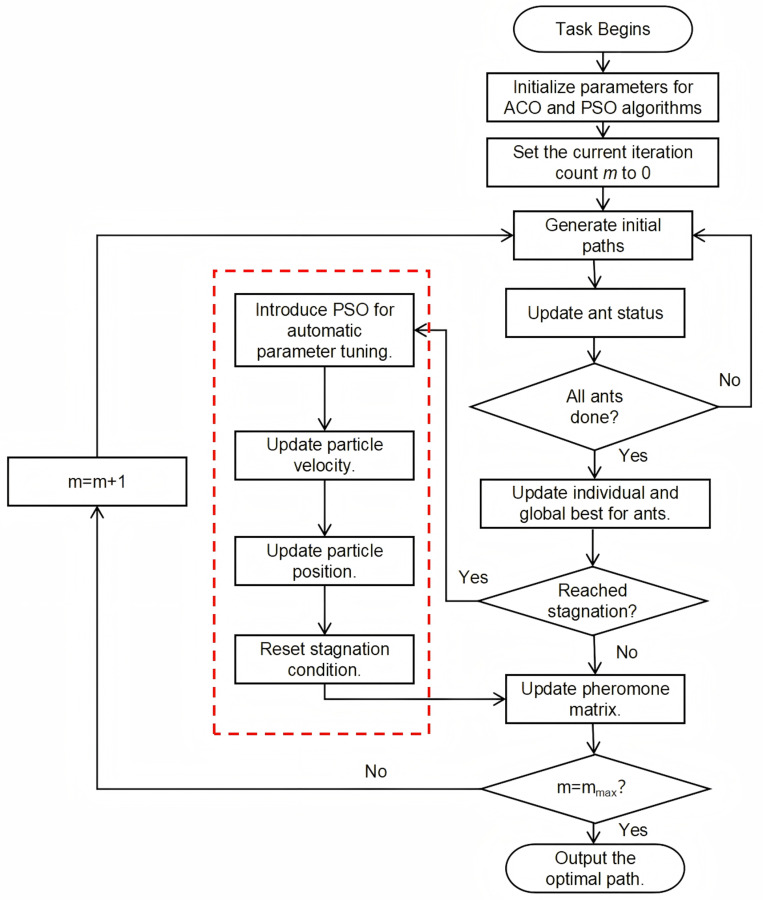
IACO algorithm flowchart.

During the iteration process of the algorithm, static parameters are used for optimization in the initial phase to ensure gradual convergence towards the objective. In the later stages, parameters are dynamically adjusted through PSO, effectively mitigating premature convergence. Each ant’s path represents the transportation route of container trucks. The total operational cost of the truck fleet is used as the fitness function, and in conjunction with the actual conditions of the truck scheduling under the appointment system, the pheromone matrix and state transition rules are redesigned to better simulate the actual scheduling problem and optimize the total fleet cost. The process of the improved algorithm is shown in Fig 3.

#### Pheromone update rule.

Unlike the traditional Vehicle Routing Problem with Time Windows (VRPTW), which typically involves single-point transport, container trucks in this context are required to perform bidirectional transport between multiple depots and terminals. Therefore, pheromones are set as a four-dimensional matrix, representing the sequence of external truck dispatches, time window, origin, and destination, respectively. Pheromones are updated after all ants complete a single iterative process and an additional pheromone update is applied to the best-performing ant identified so far. The pheromone update rules for both container pickup and delivery scenarios are the same. The following are the formulas for updating the pheromone trail related to delivery tasks performed by terminal trucks:


$$\tau_{\text{ijntk}}\text{(m+1)=}\left[\frac{\text{(1-}\rho^{\frac{m+c}{c}})\tau_{\text{ijntk}}(m)f_{avg}}{f_{best}}\right]+\Delta \tau (m)X_{\text{ijntk}}+e\Delta \tau '(m)X_{\text{ijntk}}$$ (15)

$$\Delta \tau (m)=\frac{Q}{\sqrt{f}}$$ (16)

Where, *ρ* represents the pheromone evaporation rate, *τ*_*ijntk*_*(m)* denotes the pheromone concentration of truck *k* delivering from depot *i* to terminal *j* during time period *t* at iteration *m*, c is a constant, *f*_*avg*_ is the average fitness of all current feasible solutions, and *f*_*best*_ is the fitness of the best solution found so far, which corresponds to the minimum cost of the truck companies. *Δτ(m)* is the amount of pheromone increment for ants. Q is the pheromone increment constant. *f* is the fitness function value for the ant. *X*_*ijntk*_ is the decision variable indicating whether truck *k* delivers from depot *i* to terminal *j* during time period *t*, and *e* represents the pheromone weight of elite ants. *Δτ’(m)* is defined similarly to *Δτ(m)*, but *f* corresponds to the fitness function of elite ants. To prevent excessive pheromone values that may lead to local optima, upper and lower limits are set for the pheromone levels.

The IACO algorithm could reduce the pheromone evaporation rate in the early iteration stage and dynamically adjust the evaporation rate according to the number of iterations. The algorithm enhances the search capability in the initial stages and thus avoids premature convergence.

#### State transition rule.

The state transition rule in this study is determined by the roulette method. The probability of selecting a particular path is influenced by pheromone concentration along with the heuristic factor, determinants of time window width, and the priority level of loaded versus empty trips. It should be noted that the probability of a truck selecting a customer is not related to the path length but is instead dependent on the degree of time deviation. The formulas for the state transition rule are as follows:


$$p_{ijntk}=\{\begin{array}{c} \frac{{\tau^{\alpha }}_{ijntk}{\eta^{\beta }}_{ijntk}w_{ij}d_{ij}}{\sum_{p\in P_{ipntk}}{\tau^{\alpha }}_{ipntk}{\eta^{\beta }}_{ipntk}w_{ij}d_{ij}},p\in P_{ipntk}\\ 0,p\notin P_{ipntk} \end{array}$$ (17)

$${\eta^{\beta }}_{ijntk}=\frac{1}{S_{ijntk}}$$ (18)

$$S_{ijntk}=\{\begin{array}{c} a_{1}P_{1}\left({t^{r}}_{ij}-t_{a}\right)+b_{1},t_{a}\in \left({t^{r}}_{ij}-t_{e},{t^{r}}_{ij}\right)\\ 1,t_{a}\in \left({t^{r}}_{ij}-{t^{d}}_{ij}\right)\\ a_{2}P_{2}\left(t_{a}-{t^{d}}_{ij}\right)+b_{2},t_{a}\in \left({t^{d}}_{ij},{t^{d}}_{ij}+t_{1}\right) \end{array}$$ (19)

$$w_{ij}={\text{t}}_{ij}^{d}-{\text{t}}_{ij}^{r}$$ (20)

$$d_{ij}= \{\begin{array}{c} \frac{1}{D_{ij}},\text{When transporting empty from yard i to terminal j}\\ \frac{\lambda }{D_{ij}}�\text{When transporting loaded from yard i to terminal j} \end{array}$$ (21)

Eq (17) is the state transition formula, where *P*_*ijntk*_ is the probability that truck *k* moves from depot *i* to terminal *j* in the *nth* operation during time period *t*. *τ*_*ijntk*_ and *η*_*ijntk*_ are the pheromone factor and heuristic factor respectively. *S*_*ijntk*_ is the time offset factor. Eqs (18 and 19) represent the degree of time offset, influencing the magnitude of heuristic factors, where *a*_*1*_, *a*_*2*_, *b*_*1*_, and *b*_*2*_ are constant parameters. Eq (20) defines the duration of the time window. Eq (21) introduces factors for overload and empty load effects, where *λ* is a constant parameter.

#### PSO velocity and position update equations.

Ant Colony Optimization mimics the natural phenomenon where ants use pheromones to select paths during foraging. In the algorithm, ants are more likely to deposit pheromones on paths with higher pheromone concentrations, reinforcing these paths through positive feedback. By adjusting two key parameters, pheromone weight (*α*) and heuristic weight (*β*), the algorithm strikes a balance between exploring new paths and exploiting existing good paths. To further enhance the performance of ACO, this study incorporates PSO to automatically adjust the heuristic and pheromone factors in ACO. PSO dynamically optimizes the parameters, improving the algorithm’s adaptability and robustness to different problem instances, thereby enhancing the quality of the solutions. The following are the updated equations for particle velocity and position in PSO:


$$V_{id}^{k}=WV_{id}^{k-1}+c_{1}r_{1}(pbest_{id}-x_{id}^{k-1})+c_{2}r_{2}(gbest_{id}-x_{id}^{k-1})$$ (22)

$$X_{id}^{k}=X_{id}^{k-1}+V_{id}^{k}$$ (23)

Eq (22) indicates that the d-dimensional velocity of particle *i* in the *k*th iteration consists of three parts, i.e., the particle’s previous velocity, the cognitive component, and the social component. The cognitive component is the difference between the particle’s current position and the best position found during the iteration. The social component is the distance between the particle’s current position and the best position found by the entire swarm. Eq (23) indicates that the updated position of the particle is the sum of its previous position and the updated velocity of the particle.

#### Tabu list setup.

The truck scheduling problem under the appointment mechanism can be viewed as a variant of the Vehicle Routing Problem with Time Windows, but it differs from the traditional vehicle scheduling problem. Specifically, trucks are allowed to make multiple round trips if customer demands are not met. After each delivery or pickup task at a yard or terminal, the demand at the customer point decreases. When the demand at a customer point reaches zero, the point is added to the tabu list, indicating that no further tasks will be assigned to it. The algorithm continues simulating the scheduling process until all customer demands are satisfied. This process is achieved through iterative cycles until all tasks are completed.

#### Pseudocode.

**Fig 4:**
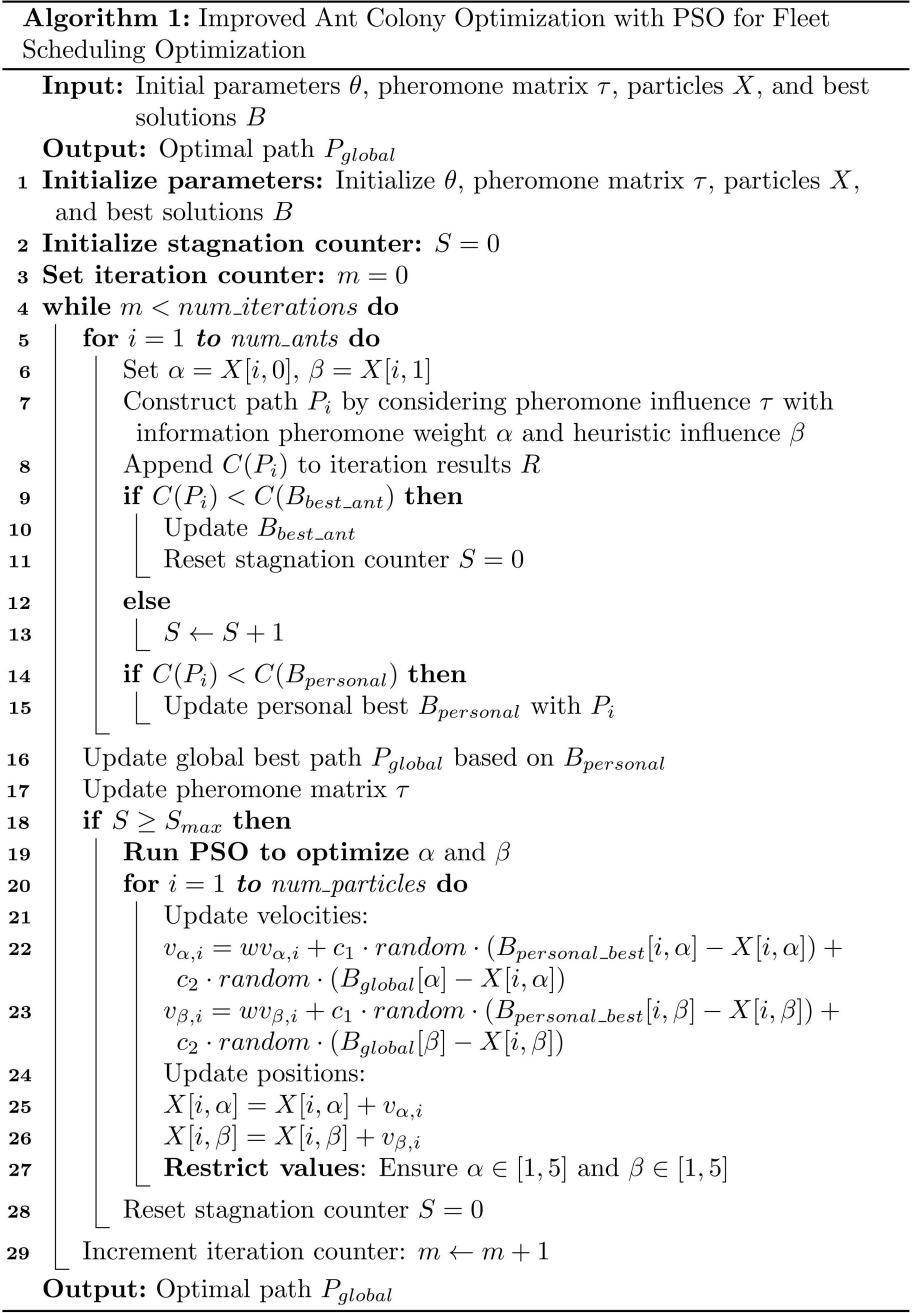
IACO pseudo code.

The IACO algorithm proposed in this paper is an improvement based on the ant colony optimization algorithm, combined with particle swarm optimization for automatic parameter tuning. The pseudocode is shown in Fig 4.

## Experimental design and results

### Experimental setup

This study aims to validate the effectiveness of the proposed external truck scheduling model and the improved Ant Colony Optimization algorithm. To this end, a case study is constructed, simulating the task of a trucking company responsible for handling container delivery and pickup tasks between a terminal and six external yards within a 24-hour task cycle. The time information for a delivery task from external yard *i* to terminal *j* can be represented as follows, where *t*^*r*^_*ij*_ and *t*^*d*^_*ij*_ denote the start and end times of the delivery task time window, respectively, with the time unit in minutes. *Q*_*ij*_ and *Q*_*ji*_ represent the number of delivery tasks from yard *i* to terminal *j* and the number of pickup tasks from terminal *j* to yard *i*, respectively.

Detailed information on the container delivery tasks between the terminal and each external yard is presented in Table 2. This table provides a comprehensive list of the task times and task quantities between each yard and the terminal.

**Table 2:** Container delivery and pickup task information table.

	*t*^ r^_ij_(min)	*t*^ d^_ij_(min)	*Q*_ij_	*Q*_ji_
Depot 2	0	720	63	50
Depot 3	240	840	51	60
Depot 4	960	1,440	43	40
Depot 5	0	360	36	45
Depot 6	720	1,080	31	36
Depot 7	720	1,440	72	65

The TAS divides the 24-hour day into 12 periods, each lasting 2 hours. The maximum number of trucks (*R*_*t*_) that the terminal can accept during each period is shown in Table 3.

**Table 3:** The maximum number of trucks that each terminal can accept during different periods.

	*R*_*1*_	*R*_*2*_	*R*_*3*_	*R*_*4*_	*R*_*5*_	*R*_*6*_	*R*_*7*_	*R*_*8*_	*R*_*9*_	*R*_*10*_	*R*_*11*_	*R*_*12*_
Terminal 1	41	39	45	42	40	31	37	35	37	41	39	43

The route distances between each external yard and the terminal are shown in Table 4.

**Table 4:** Distances between each depot and the terminal.

	Terminal 1	Depot 2	Depot 3	Depot 4	Depot 5	Depot 6	Depot 7
Terminal 1	0	40	43	30	30	16	21
Depot 2	40	0	44	15	46	24	30
Depot 3	43	44	0	31	16	42	23
Depot 4	30	15	31	0	32	18	16
Depot 5	30	46	16	32	0	46	42
Depot 6	16	24	42	18	46	0	19
Depot 7	21	30	23	16	42	19	0

The full-load speed of the truck is 40 km/h, while the empty-load speed is 60 km/h. The fuel consumption costs per kilometer for full-load and empty-load operations are 2.8 and 1.76 RMB/km, respectively. The idling fuel consumption cost is 6.4 RMB/h. The truck driver’s salary and depreciation costs are 400 RMB/day, and the waiting time at the yard is 120 minutes. The remaining key parameters are shown in Table 5.

**Table 5:** Truck fleet pickup task appointment information table.

parameter	value	parameter	value
Depot waiting time (t_w_)	3	Elastic time window front-end floating time (t_e_)	15
Average waiting time at the terminal gate (t_2_)	5	Elastic time window backend floating time (t_l_)	60
Terminal operation time (t_3_)	2.5	Early arrival penalty factor (p_1_)	0.2
Average handling time at the depots (t_1_)	3.5	Late penalty coefficient (p_2_)	2

In this study, we configured the parameters for the IACO algorithm as follows: the ant colony consists of 10 ants, with pheromone increments set to 100 for ordinary ants and 1,000 for elite ants to reinforce optimal paths. The constant c is set to 5,000 to balance pheromone and heuristic information. The pheromone concentration is restricted between 1 and 15 to maintain diversity, while the weights for the heuristic and pheromone factors are adjusted between 1 and 5 to balance exploration and exploitation. In the Particle Swarm Optimization algorithm, the initial velocity inertia weight is set to 0.8, and the learning factor is set to 1.5 to optimize individual and group learning. These parameter settings are designed to enhance the performance of the algorithm and ensure its effectiveness in solving optimization problems.

### Result analysis

#### The impact of different evaporation rates on the performance of IACO.

In the Ant Colony Optimization algorithm, the pheromone evaporation rate is a critical parameter that influences the convergence speed of the algorithm and its ability to avoid local optima. By simulating the natural dissipation of pheromones in nature, the evaporation rate helps to reduce outdated pheromones, making space for the deposition of new pheromones, and thereby promoting the exploration of better paths. Proper adjustment of the evaporation rate plays a key role in balancing global search and local exploitation, thus optimizing the performance of the algorithm.

**Fig 5:**
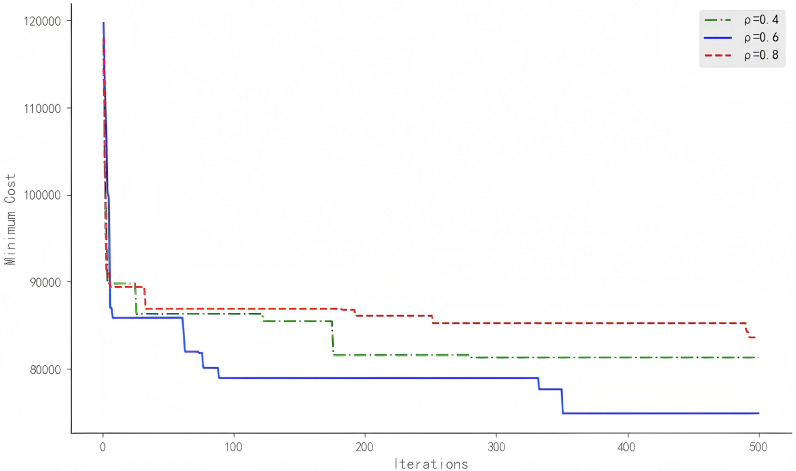
Results of different pheromone evaporation rates in the IACO algorithm.

To determine the optimal value of the pheromone evaporation rate, several experiments with evaporation rates set at 0.4, 0.6, and 0.8 were conducted. Each experiment was carried out under the same initial conditions and parameter settings to ensure the comparability and reliability of the results. By comparing the experimental outcomes at different evaporation rates, we can analyze the impact of the evaporation rate on the performance of the improved ant colony algorithm and select the optimal evaporation rate for further analysis. Fig 5 shows the iterative results of the IACO algorithm under different pheromone evaporation rates.

It is evident from the figure that the algorithm performs best with an evaporation rate of 0.6. Although the convergence speed of the algorithm is relatively slow in the early stages under an evaporation rate of *ρ* = 0.6, the algorithm effectively prevents excessive accumulation of pheromones and avoids getting trapped in local optima as the number of iterations increases. This is because a moderate evaporation rate results in a more balanced distribution of pheromones across paths, thereby enhancing the algorithm’s global search capability.

In contrast, with an evaporation rate of *ρ* = 0.4, the slower evaporation rate causes excessive accumulation of pheromones on paths, which makes it easier for the algorithm to get stuck in local optima and difficult to escape from. Although the algorithm converges faster in the early stages, the overall optimization performance is poor.

Conversely, with an evaporation rate of *ρ* = 0.8, the pheromone evaporation is too rapid, leading to minimal accumulation of pheromones on good paths, which impairs the algorithm’s ability to recognize high-quality paths. This causes the algorithm to tend towards random search and results in poorer optimization performance. While a high evaporation rate can prevent excessive accumulation, it also diminishes the guiding effect of pheromones, making the search process less directed.

Considering the experimental results under different evaporation rates, it is concluded that the improved ant colony algorithm performs best with an evaporation rate of *ρ* = 0.6. At this rate, the algorithm achieves optimal optimization performance while maintaining global search capability and avoiding local optima.

These results indicate that a moderate pheromone evaporation rate is crucial for improving the performance of the ant colony algorithm. Choosing an appropriate evaporation rate not only enhances the convergence speed but also strengthens global search ability, providing effective solutions to complex optimization problems. Therefore, in subsequent experiments, we used an evaporation rate of 0.6 and compared the performance of other algorithms under the same evaporation rate.

#### Comparative analysis of different algorithms under the same evaporation rate.

Based on the improved ant colony algorithm for the scheduling scheme under the reservation mechanism, the traditional ant colony algorithm and the Variable Neighborhood Search(VNS) algorithm were designed for the same scheduling scenario. For ease of comparison, the data in Table 6 are all iteration results with *ρ* = 0.6, including the minimum cost, average cost, number of vehicles, and overload rate.

**Table 6:** Comparison of results.

Experimental Results	Lowest cost	Average cost	Number of vehicles	Overload rate
ACO	84,101	111,510	25	60.1%
VNS	77,222	121,764	22	68.1%
IACO	74,887	103,313	21	71.2%

**Fig 6:**
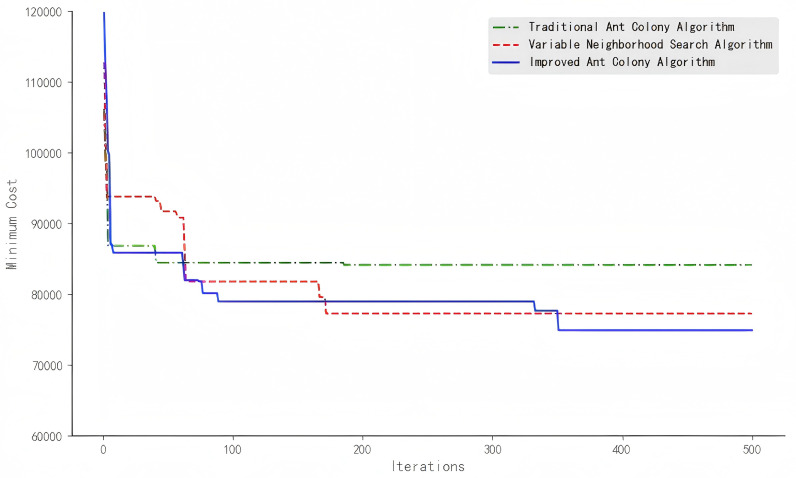
Iterative results of different algorithms.

**Fig 7:**
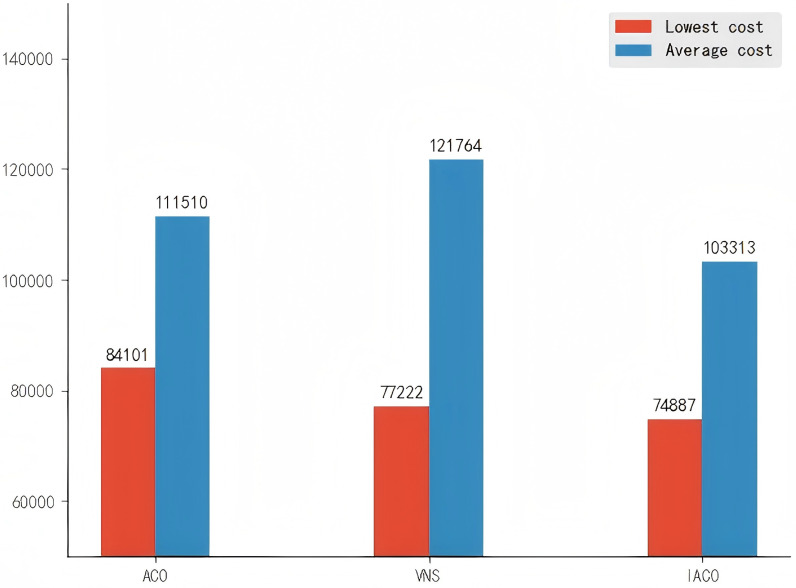
Minimum cost and average cost of different algorithms.

From Table 6, it can be observed that the IACO algorithm yields a scheduling solution with a cost reduction of 10.96% and 3.02% compared to the traditional ant colony algorithm and the VNS algorithm, respectively. Additionally, the average cost after iterations with the improved ant colony algorithm is 7.35% and 15.15% lower than that of the traditional ant colony algorithm and the VNS algorithm, respectively. It can be seen from Figs 6 and 7 that as the number of iterations increases, the cost of the optimal scheduling solution using the improved ant colony algorithm shows less fluctuation. Due to the higher pheromone evaporation rate and the constant increase of pheromones by elite ants, the scheduling solution approaches the optimal solution early in the iterations. Although there is a tendency to get trapped in local optima at certain points, the results eventually converge due to adjustments made by the PSO algorithm. After iterations, the final best cost is 74,887 RMB.

It can also be seen from Table 6 that the optimal scheduling solution produced by the improved ant colony algorithm has an overload rate of approximately 71.2%. Under the same conditions, this is superior to the results of the traditional ant colony algorithm and the VNS algorithm. In port operations, the overload rate typically ranges from 50% to 80%. A higher overload rate indicates more efficient utilization of logistics resources such as vehicles and drivers, thereby improving loading and unloading efficiency, reducing the mileage of transport vehicles, and lowering transportation costs. The results demonstrate that the improved ant colony algorithm not only excels in cost optimization but also significantly enhances resource utilization and operational efficiency. By achieving a higher overload rate, the improved algorithm can more effectively organize and schedule vehicles, allowing each trip to carry more goods, thereby reducing empty runs and repetitive transportation.

Regarding the number of trucks required for the optimal scheduling solution, the improved ant colony algorithm consistently uses fewer trucks than the traditional ant colony algorithm and the VNS algorithm. For a truck fleet, the number of trucks is limited; therefore, using fewer vehicles means that the remaining vehicles can perform additional tasks, thus increasing dispatch efficiency. The results indicate that the improved ant colony algorithm not only performs excellently in optimizing costs and improving overload rates but also significantly reduces the number of trucks required. This means that under the same transportation demand, the improved algorithm can schedule vehicles more effectively, maximizing fleet resource utilization. Fewer vehicle requirements not only reduce scheduling difficulty but also provide the fleet with greater flexibility to handle unexpected situations or other transportation tasks.

#### Compared with manual scheduling.

Traditional truck companies typically rely on manual experience for scheduling during pickup and delivery tasks, similar to a greedy strategy, with a primary focus on heavy-load transport. In most cases, schedulers will select the nearest node among all available heavy-load transport nodes at the current time as the destination. If no heavy-load nodes are available, the vehicle will enter a waiting state and attempt the transportation task again after a certain period, until all transportation tasks are completed. A comparison of the results between the IACO algorithm and the traditional scheduling approach is shown in Table 7.

**Table 7:** Comparison of Manual Scheduling Results.

Algorithm	Total Cost	Number of vehicles	Overload rate
Manual Scheduling	78,735	29	68.4%
IACO	74,887	21	71.2%

The traditional scheduling approach relies on the experience and intuition of the scheduler, which may not fully consider all variables, leading to suboptimal resource utilization. As a result, manual scheduling tends to result in lower operational costs and fewer trucks compared to the IACO algorithm. However, in terms of other metrics, the manual scheduling approach does not show a significant decrease in heavy-load transport rates. This is mainly due to its reliance on a greedy strategy. While this approach can obtain relatively good solutions in the short term, the scheduler’s manual experience struggles to comprehensively consider multiple influencing factors.

In contrast, the IACO algorithm, through systematic calculations and simulations, can evaluate the relationships between various variables more comprehensively, leading to more efficient resource allocation. The algorithm combines ant colony optimization with other heuristic techniques, using information sharing and adaptive mechanisms to adjust scheduling plans in real time, making it more adaptable to complex logistics environments than traditional scheduling.

#### Result statistics.

Table 8 shows the optimal scheduling order for each truck obtained by the improved ant colony algorithm. The left side of the table lists the required number of trucks, while the right side shows the specific scheduling routes for each truck, where 1 represents the terminal, and 2 to 7 represent different port yard numbers.

**Table 8:** Optimal scheduling sequence of IACO algorithm.

Container truck number	Container truck operation sequence
1	5, 1, 2, 1, 2, 1, 2, 1, 2, 1, 2, 1, 2, 1, 2, 1, 3, 1, 2, 1, 2, 1, 2, 1, 2, 1, 2, 1, 2, 1, 2, 1, 2, 1, 2, 1, 2, 1, 2, 1, 2, 1, 2, 1, 7, 1, 7, 1, 7, 1, 7, 1, 7, 1, 7, 1, 7, 1, 7, 1, 7, 1, 7, 1, 2, 1, 7, 1, 2, 6, 4, 5
2	2, 1, 2, 1, 2, 1, 2, 1, 2, 1, 2, 1, 2, 1, 2, 1, 2, 1, 2, 1, 2, 1, 2, 1, 2, 1, 2, 1, 2, 1, 2, 1, 2, 1, 2, 1, 2, 1, 2, 1, 2, 1, 2, 1, 2, 1, 2, 1, 2, 1, 7, 4, 1, 6, 1, 7, 1, 4, 1, 7, 1, 2, 1, 3, 5, 4, 1, 2, 3, 5, 7, 2
3	2, 1, 2, 1, 2, 1, 5, 1, 7, 1, 7, 5, 1, 7, 5, 1, 5, 4, 3, 1, 7, 3, 1, 7, 1, 7, 1, 4, 1, 7, 1, 7, 1, 4, 1, 7, 1, 4, 1, 7, 1, 2
4	2, 1, 7, 2, 1, 5, 1, 5, 1, 7, 2, 1, 4, 3, 1, 7, 5, 4, 2, 1, 5, 6, 1, 5, 2, 1, 7, 1, 7, 1, 7, 1, 7, 1, 7, 1, 7, 1, 4, 1, 7, 1, 7, 1, 7, 2
5	2, 1, 5, 1, 5, 1, 3, 1, 3, 1, 3, 1, 7, 5, 3, 1, 7, 1, 7, 1, 6, 1, 7, 1, 7, 1, 7, 1, 7, 1, 7, 1, 7, 1, 7, 1, 7, 1, 7, 1, 7, 1, 2
6	2, 1, 5, 1, 7, 1, 7, 2, 1, 7, 5, 1, 7, 1, 3, 1, 3, 1, 7, 1, 6, 1, 6, 1, 6, 1, 6, 1, 6, 1, 7, 1, 7, 1, 7, 1, 4, 1, 3, 5, 2
7	2, 1, 5, 1, 3, 4, 2, 1, 7, 2, 1, 3, 1, 6, 4, 5, 2, 1, 6, 1, 4, 3, 1, 4, 1, 7, 1, 5, 7, 1, 5, 4, 1, 7, 1, 7, 1, 2
8	2, 1, 7, 3, 1, 3, 1, 5, 1, 3, 1, 3, 1, 3, 1, 6, 1, 7, 1, 7, 1, 6, 1, 4, 1, 4, 1, 4, 1, 7, 1, 5, 7, 2
9	2, 1, 3, 6, 2, 1, 3, 1, 3, 1, 3, 1, 3, 1, 5, 4, 1, 3, 1, 4, 1, 6, 1, 4, 1, 4, 1, 4, 2, 7, 2
10	2, 1, 5, 1, 5, 1, 3, 1, 5, 1, 3, 1, 3, 1, 6, 1, 6, 1, 6, 1, 6, 1, 6, 1, 4, 1, 4, 1, 5, 3, 6, 2
11	5, 1, 6, 7, 1, 3, 1, 5, 1, 3, 1, 3, 1, 6, 1, 6, 1, 6, 1, 4, 1, 5, 3, 5, 1, 4, 1, 6, 1, 3, 2, 7, 1, 5
12	5, 1, 5, 1, 5, 1, 3, 1, 3, 1, 3, 1, 6, 1, 6, 1, 6, 1, 4, 1, 4, 1, 4, 1, 6, 2, 3, 7, 1, 5, 3, 5
13	5, 1, 5, 1, 3, 1, 3, 1, 3, 1, 4, 5, 3, 1, 6, 1, 6, 1, 4, 1, 6, 1, 6, 4, 1, 3, 4, 1, 7, 2,5
14	1, 5, 1, 5, 1, 5, 1, 5, 1, 3, 1, 3, 1, 4, 1, 3, 1, 4, 3, 4, 1, 6, 1, 4, 1, 4, 1, 3, 7, 1
15	5, 1, 5, 1, 3, 1, 3, 1, 3, 1, 3, 1, 3, 1, 4, 6, 1, 4, 1, 4, 1, 4, 1, 4, 1, 6, 2, 7, 5
16	5, 1, 5, 1, 3, 1, 3, 1, 3, 1, 6, 5, 2, 6, 1, 6, 1, 5, 3, 6, 1, 6, 1, 4, 1, 4, 1, 4, 1, 2, 1, 5
17	5, 1, 5, 1, 5, 1, 5, 1, 5, 7, 2, 5, 7, 2, 3, 1, 5, 7, 1, 6, 1, 5, 4, 1, 4, 1, 5, 4, 1, 5, 2, 7, 1, 4, 1, 2,5
18	1, 5, 1, 4, 6, 2, 7, 1, 3, 1, 3, 1, 3, 1, 3, 1, 3, 1, 5, 3, 7, 1, 3, 2, 5, 3, 5, 2, 6, 2, 3, 5, 2, 3, 5, 7, 1
19	5, 1, 3, 7, 5, 3, 7, 2, 7, 5, 3, 5, 3, 2, 5, 2, 7, 1, 3, 5, 7, 5, 7, 1, 3, 4, 6, 4, 1, 3, 5, 2, 7, 1, 5, 4, 1, 5, 4, 1, 2,5
20	1, 3, 5, 4, 2, 7, 2, 7, 1, 5, 3, 4, 2, 7, 3, 5, 7, 2, 7, 2, 7, 2, 5, 7, 1, 3, 2, 7, 1, 3, 2, 7, 1, 3, 5, 4, 1, 7, 1, 7, 1, 7, 1, 7, 1, 7, 1
21	5, 2, 7, 2, 4, 3, 7, 5, 3, 7, 6, 4, 5, 7, 3, 2, 7, 5, 3, 2, 7, 1, 7, 1, 7, 1, 7, 1, 7, 1, 7, 1, 7, 1, 7, 1, 7, 1, 7, 1, 7, 5, 4, 1,5

**Fig 8:**
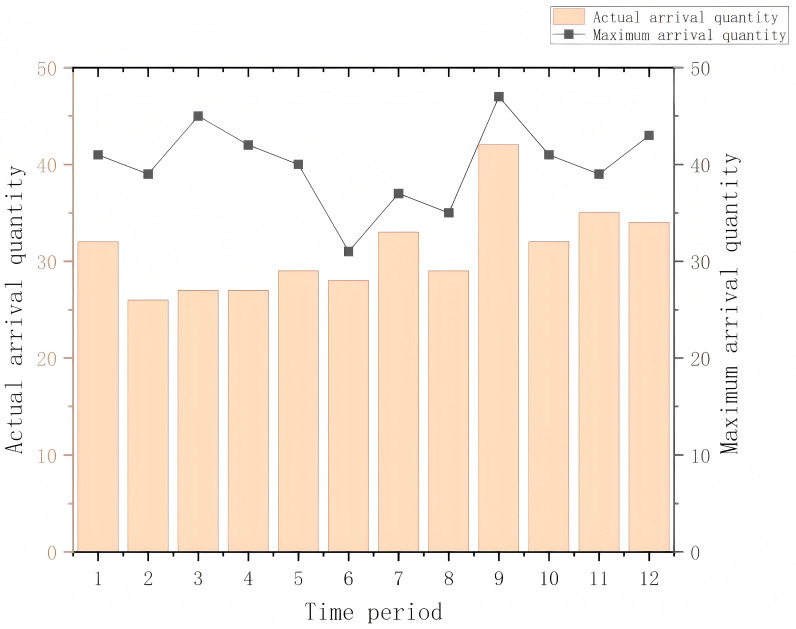
Number of vehicles arriving at the terminal during different periods.

Fig 8 illustrates the comparison between the actual arrival quantity and the maximum possible arrival quantity at the terminal gate across 12 consecutive time periods. The orange bars represent the actual arrival quantities in each time period, while the black line indicates the maximum possible arrival quantity. The actual arrival quantity remains relatively stable across the time periods, with the improved ant colony optimization algorithm ensuring a more evenly distributed arrival of trucks across the time slots. This effectively prevents the over-concentration of truck arrivals during certain periods. Additionally, the number of trucks arriving in each time slot does not exceed the maximum reservation capacity of the gate, further validating that the algorithm not only meets system constraints but also optimizes the scheduling plan, ensuring smooth terminal operations.

In summary, in the truck scheduling under the appointment mode, the improved ant colony optimization algorithm, which integrates the advantages of the particle swarm optimization algorithm, outperforms traditional ant colony optimization, variable neighborhood search, and manual scheduling. In terms of minimum cost, the improved algorithm reduces the cost by 10.96% and 3.02% compared to traditional ant colony optimization and variable neighborhood search, respectively, and by 4.89% compared to manual scheduling. For average cost, the improved algorithm decreases the cost by 7.35% and 15.15% compared to traditional ant colony optimization and variable neighborhood search, respectively. Moreover, in terms of the number of vehicles required and the heavy-load rate, the improved ant colony optimization algorithm also performs exceptionally well.

The improved ant colony algorithm not only effectively reduces the waiting time for vehicles at the depot and terminal but also greatly enhances dispatch efficiency. By optimizing the scheduling scheme, this algorithm better coordinates and utilizes limited fleet resources, thereby improving the overall operational efficiency of the logistics system. Overall, the improved ant colony algorithm provides a highly efficient and cost-effective scheduling solution for ports and logistics centers, with broad application potential.

## Conclusion

This paper focuses on optimizing the scheduling model for container truck fleets under a reservation mechanism, specifically targeting the delivery and pickup of containers. The goal is to minimize the operational costs of the fleet while meeting the demands of the terminal and depots within the specified time frame. A vehicle scheduling optimization model is designed, and both the IACO algorithm and PSO algorithm are proposed for solving the model. The performance of these algorithms is validated through comparative experiments. The main findings of the paper are as follows:

(1) By analyzing the workflow of container delivery and pickup between multiple depots and a single terminal under the reservation mode, and considering the reservation quotas and flexible time windows, the paper aims to minimize fleet operational costs. Based on realistic assumptions, a scheduling optimization model for container delivery and pickup between depots and the terminal was constructed. The final result is the operational sequence for the container truck fleets of each depot.
(2) Given the complexity of the model and the numerous factors involved, obtaining an optimal solution is challenging. The paper designs an improved ant colony algorithm to solve the model. This algorithm modifies the pheromone update rules from the traditional ant colony algorithm and introduces a time window width influence factor and time deviation degree in the state transition rules. It also combines PSO to automatically adjust heuristic influence factors and pheromone influence factors, thereby improving the ability to escape local optima. The results show that this algorithm significantly enhances optimization capability and stability compared to the traditional ant colony algorithm and the VNS algorithm.
(3) To validate the model and solution algorithm, a set of experiments was conducted. The comparison results show that, in terms of minimum cost, the IACO algorithm reduces the cost by 10.96% and 3.02% compared to the traditional ACO and VNS algorithms, respectively, and by 4.89% compared to manual scheduling. Regarding the average cost, the improved algorithm decreases the cost by 7.35% and 15.15% compared to the traditional ACO and VNS algorithms, respectively. Additionally, improvements were observed in the number of vehicles used and the load rate.

Although this study successfully reduced fleet operational costs and partially addressed port congestion issues, further work is needed to improve terminal dispatch efficiency. First, additional factors affecting container truck scheduling can be considered in further work to better reflect real-world situations, such as vehicle speeds during peak hours, truck no-shows, and gate queuing patterns. Second, the model and the algorithms could be further ameliorated to better reduce fleet costs and improve dispatch efficiency.

## Supporting information

S1 File Code. (ZIP)

S2 File Date. (ZIP)
