# Supplementary material for: Optimization of external container delivery and pickup scheduling based on appointment mechanism
Source: PLoS One. 2025 Feb 21;20(2):e0318606. doi: 10.1371/journal.pone.0318606 (PMC11844885; doi:10.1371/journal.pone.0318606)
Supplement: S1 File — (ZIP) [file pone.0318606.s001.zip › S1 file/Code/README.docx]

**Runtime Requirements:**

To execute the provided code, the following Python packages are required:

math: A built-in Python module for mathematical operations.

numpy: A fundamental package for scientific computing with Python.

random: A Python module for generating random numbers.

matplotlib: A plotting library for creating static, animated, and interactive visualizations in Python.

**Setup Instructions:**

Verify that the necessary packages are installed in your Python environment. If any are missing, you can install them using pip:

pip install numpy matplotlib

Note: The math and random modules are part of Python's standard library and do not require separate installation.

Arrange your files by placing the code file and all related files in the same folder.If you are using PyCharm as your Python Integrated Development Environment (IDE), you will need to put these files in the root directory of PyCharm.

To run the code, open the script in your preferred Python Integrated Development Environment (IDE) or execute it from the command line.

**Running the Code:**

After the setup, you can run the code by opening the script.
